# Non-myogenic mesenchymal cells contribute to muscle degeneration in facioscapulohumeral muscular dystrophy patients

**DOI:** 10.1038/s41419-022-05233-6

**Published:** 2022-09-16

**Authors:** Lorena Di Pietro, Flavia Giacalone, Elvira Ragozzino, Valentina Saccone, Federica Tiberio, Marco De Bardi, Mario Picozza, Giovanna Borsellino, Wanda Lattanzi, Enrico Guadagni, Sara Bortolani, Giorgio Tasca, Enzo Ricci, Ornella Parolini

**Affiliations:** 1 Dipartimento di Scienze della Vita e Sanità Pubblica, Università Cattolica del Sacro Cuore, Rome, Italy; 2 Fondazione Policlinico Universitario A. Gemelli IRCSS, Rome, Italy; 3 Neuroimmunology Unit, IRCCS Santa Lucia Foundation, Rome, Italy; 4 Unità Operativa Complessa di Neurologia, Fondazione Policlinico Universitario A. Gemelli IRCCS, Rome, Italy; 5 Dipartimento di Neuroscienze, Università Cattolica del Sacro Cuore, Rome, Italy

**Keywords:** Mechanisms of disease, Stem-cell differentiation

## Abstract

Muscle-resident non-myogenic mesenchymal cells play key roles that drive successful tissue regeneration within the skeletal muscle stem cell niche. These cells have recently emerged as remarkable therapeutic targets for neuromuscular disorders, although to date they have been poorly investigated in facioscapulohumeral muscular dystrophy (FSHD). In this study, we characterised the non-myogenic mesenchymal stromal cell population in FSHD patients’ muscles with signs of disease activity, identified by muscle magnetic resonance imaging (MRI), and compared them with those obtained from apparently normal muscles of FSHD patients and from muscles of healthy, age-matched controls. Our results showed that patient-derived cells displayed a distinctive expression pattern of mesenchymal markers, along with an impaired capacity to differentiate towards mature adipocytes in vitro, compared with control cells. We also demonstrated a significant expansion of non-myogenic mesenchymal cells (identified as CD201- or PDGFRA-expressing cells) in FSHD muscles with signs of disease activity, which correlated with the extent of intramuscular fibrosis. In addition, the accumulation of non-myogenic mesenchymal cells was higher in FSHD muscles that deteriorate more rapidly. Our results prompt a direct association between an accumulation, as well as an altered differentiation, of non-myogenic mesenchymal cells with muscle degeneration in FSHD patients. Elucidating the mechanisms and cellular interactions that are altered in the affected muscles of FSHD patients could be instrumental to clarify disease pathogenesis and identifying reliable novel therapeutic targets.

## Introduction

Facioscapulohumeral muscular dystrophy (FSHD) is one of the most prevalent muscular dystrophies worldwide and is characterised by a slowly progressive muscle weakness and atrophy [1]. The most prevalent form of FSHD (FSHD1) is associated with a contraction of the D4Z4 macrosatellite region on chromosome 4q35, leading to the inappropriate transcription of the *double homeobox 4* (*DUX4*) gene [2, 3], which is normally silenced in skeletal muscle. The inappropriate expression of DUX4, followed by the activation of its target genes in the skeletal muscle of FSHD patients, represent crucial events likely driving muscle degeneration in FSHD. Nonetheless, the exact molecular and cellular mechanisms involved in the pathogenesis of this progressive degeneration have yet to be clarified. Notably, a distinctive hallmark of FSHD is the asynchronous progression of muscle degeneration, which involves single muscles with apparently sparing adjacent muscles in the same patient.

Skeletal muscle possesses the peculiar ability to regenerate and harbours a stem cell niche in which different cell types actively participate and collaborate to drive successful tissue regeneration. The niche environment includes satellite cells, which represent the self-renewing muscle stem cells (MuSCs) [4], and committed progenitors (i.e., myoblasts). Once activated, these cells undergo myogenic differentiation leading to the formation of new muscle fibres. The muscle niche also includes immune cells, non-satellite cells with myogenic potential and non-myogenic mesenchymal stromal cells [5–7]. The latter were originally referred to as fibro-adipogenic progenitors (FAPs) [6], while in recent years, mainly thanks to the results obtained with innovative single-cell profiling techniques, the definition of these cells has changed. It is currently clear that this population consists of heterogeneous subtypes [8, 9], spanning a wide progeny from progenitors to more committed cells. Based on this background and since the commonly used markers are not able to discriminate between progenitors, early committed cells, and fully differentiated stromal cells, such as fibroblasts, we will refer to these cells as non-myogenic mesenchymal cells. Non-myogenic mesenchymal cells play pivotal roles in muscle regeneration, acting as important mediators between immune cells and MuSCs, and secreting different signals able to sustain MuSC proliferation and differentiation [7, 10]. In pathologic situations, the dysregulation of non-myogenic mesenchymal cell proliferative and/or differentiative potential is responsible for muscle fibrosis and adipogenesis that impair muscle functionality [11–16]. At present, although recent evidence in an FSHD animal model suggested a possible implication of non-myogenic mesenchymal cells in the progressive fibrotic degeneration of skeletal muscle that characterises FSHD [17–19], the involvement of these cells in FSHD pathophysiology has been poorly investigated in humans.

Muscle magnetic resonance imaging (MRI) has emerged as a useful clinical tool to describe muscle involvement in muscular dystrophies, including FSHD, giving a significant contribution to the understanding of disease progression. In particular, MRI allows to evaluate the involvement of individual muscles by assessing skeletal muscle oedema, which affects signal on short-tau inversion recovery (STIR) sequences, and adipose tissue infiltration, which in turn enhances signal on T1 sequences [20–22]. The early phases of muscle damage are identifiable by an increased hyperintensity on STIR sequences, that accounts for muscle inflammation [23], even in muscles showing no signs of fat accumulation [21, 24].

The objective of our study was to characterise the non-myogenic mesenchymal stromal cell population in affected muscles of FSHD patients, compared with both control and FSHD apparently normal muscles, leveraging the detailed framework provided by MRI, to clarify their possible involvement in the pathogenesis of FSHD. Our results showed that non-myogenic mesenchymal cells isolated from FSHD muscles displayed a peculiar expression pattern of mesenchymal markers and an altered adipogenic differentiative potential compared to cells isolated from healthy muscles. We also observed an expansion of CD201^+^ and PDGFRA^+^ non-myogenic mesenchymal cells in FSHD STIR+ muscles that positively correlated with muscle fibrosis and disease progression.

## Materials and methods

### Patient enrolment and specimen collection

Twenty-seven FSHD patients with a confirmed FSHD1 diagnosis were enroled during MRI follow-up studies at Fondazione Policlinico Universitario Agostino Gemelli IRCCS. All protocols were conducted according to the European Good Clinical Practice guidelines after the approval by the Ethical Committee of the Fondazione Policlinico Universitario Agostino Gemelli IRCCS, Università Cattolica del Sacro Cuore (protocol ID 1524 and ID 3637), and upon obtaining the written informed consent. Nine control subjects were also enroled among healthy volunteers, including unaffected relatives of FSHD patients, that were age-matched [mean age controls ± standard deviation (SD): 47 ± 15 years, mean age FSHD patients ± SD: 46 ± 15 years]. Muscle tissue samples were collected through needle muscle biopsies after MRI examination. Baseline and 1-year follow-up images were evaluated using a 5-point semiquantitative scale to assess the extent of fatty replacement in single muscles on T1w sequences [25] and using a binary score (i.e., YES or NO) to judge the presence of hyperintensities on STIR sequences. Clinical and genetic data of all the subjects enroled were recorded and reported in Table 1. Muscle biopsies were simultaneously or alternatively processed for cell isolation, RNA extraction and immunofluorescence analysis.

**Table 1:** Data of enroled subjects.

Group	Patient (number)	Diagnosis	EcoRI fragment lenght	Sex	Clinical Severity score (CSS)	Age at biopsy (years)	Site of biopsy	STIR MRI signal	T1w MRI signal	Progression
**Control muscles**	CTRL#1	-	N/A	M	N/A	56	Vastus lateralis	N/A	N/A	N/A
	CTRL#2	-	N/A	M	N/A	52	Vastus lateralis	N/A	N/A	N/A
	CTRL#3	-	N/A	M	N/A	36	Vastus lateralis	N/A	N/A	N/A
	CTRL#4	-	N/A	M	N/A	57	Vastus lateralis	N/A	N/A	N/A
	CTRL#5	-	N/A	M	N/A	36	Vastus lateralis	N/A	N/A	N/A
	CTRL#6	-	N/A	M	N/A	57	Vastus lateralis	N/A	N/A	N/A
	CTRL#7	-	N/A	M	N/A	20	Vastus lateralis	N/A	N/A	N/A
	CTRL#8	-	N/A	F	N/A	70	Vastus lateralis	N/A	N/A	N/A
	CTRL#9	-	N/A	F	N/A	41	Vastus lateralis	N/A	N/A	N/A
**FSHD STIR**− **muscles**	FSHD#1	FSHD1	28	F	0	49	Vastus lateralis	NO	0	NO
	FSHD#2	FSHD1	26	M	3.5	60	Vastus lateralis	NO	2	NO
	FSHD#3	FSHD1	25	F	4	62	Vastus lateralis	NO	0	NO
	FSHD#4	FSHD1	23	M	1	34	Vastus lateralis	NO	0	NO
	FSHD#5	FSHD1	26	M	3	57	Vastus lateralis	NO	0	NO
	FSHD#6	FSHD1	20	M	4.5	32	Gastrocnemius	NO	0	NO
	FSHD#7	FSHD1	24	F	3	65	Vastus lateralis	NO	0	NO
	FSHD#8	FSHD1	24	M	3	29	Gastrocnemius	NO	0	NO
	FSHD#9	FSHD1	30	M	4	49	Vastus lateralis	NO	0	NO
	FSHD#10	FSHD1	24	M	1	37	Semimebranous	NO	0	NO
	FSHD#11	FSHD1	24	F	1.5	53	Vastus lateralis	NO	1	NO
	FSHD#12	FSHD1	36	F	4	58	Vastus lateralis	NO	2	NO
	FSHD#13	FSHD1	N/A	M	3	64	Vastus lateralis	NO	1	N/A
**FSHD STIR**+ **muscles**	FSHD#14	FSHD1	24	M	2.5	60	Vastus lateralis	YES	1	N/A
	FSHD#15	FSHD1	17	F	4.5	33	Vastus lateralis	YES	1	YES
	FSHD#16	FSHD1	22	M	3.5	47	Vastus lateralis	YES	2	N/A
	FSHD#17	FSHD1	26	M	1	22	Gastrocnemius	YES	3	YES
	FSHD#18	FSHD1	29/trisomic	M	1.5	70	Vastus lateralis	YES	2	YES
	FSHD#19	FSHD1	27	M	2.5	34	Gastrocnemius	YES	1	YES
	FSHD#20	FSHD1	27	M	2.5	27	Tibialis anterior	YES	0	N/A
	FSHD#21	FSHD1	22	M	3.5	40	Gastrocnemius	YES	1	YES
	FSHD#22	FSHD1	27	M	4	65	Gastrocnemius	YES	1	YES
	FSHD#23	FSHD1	26	M	4	62	Vastus lateralis	YES	2	N/A
	FSHD#24	FSHD1	20	M	4.5	32	Tibialis anterior	YES	1	YES
	FSHD#25	FSHD1	18	F	3	29	Gastrocnemius	YES	0	YES
	FSHD#26	FSHD1	38	M	1.5	24	Tibialis anterior	YES	0	YES
	FSHD#27	FSHD1	21	M	4	56	Tibialis anterior	YES	2	N/A

FSHD1 diagnosis was genetically confirmed by the presence of 3–9 KpnI D4Z4 repeats on the 4q35 chromosome. *M* male, *F* female, *N/A* not assessed. Progression was assessed at 1-year follow-up and reported as YES if there was an increase in T1w sequences since the time of biopsy, or NO if a worsening in T1w sequences was not registered.

### Cell isolation and culture

Upon collection, biopsies from four control, four FSHD STIR− and five FSHD STIR+ muscles were mechanically and enzymatically digested to isolate cells. Muscle specimens were washed in phosphate-buffered saline (PBS, Aurogene, Rome, Italy) supplemented with 1% antibiotics (penicillin 100 IU/ml, streptomycin 100 mg/ml, Aurogene), initially processed by manual fragmentation with scissors and subsequently digested with 2 mg of collagenase/dispase (Merck, Darmstadt, Germany) at 37 °C for 30 min. The tissue homogenates were filtered through a 40 µm cell strainer and the cells pelleted after centrifugation were resuspended in CYTO-GROW medium (Resnova, Rome, Italy). Cells were plated in a 25 cm^2^ flask coated with 0.1% Gelatin (Stem cell Technologies, Vancouver, Canada) and incubated in a humidified atmosphere at 37 °C and 5% CO_2_.

### Cell sorting and flow cytometry

Upon reaching sub-confluence, the mixed adherent cell population was sorted by FACS (MoFlo Astrios EQ HighSpeed Cell Sorter, Beckman Coulter, Brea, CA, USA) on the basis of the expression of the myogenic marker CD56 (1:50, #12-0567-42, eBioscience) [26]. Cell sorting allowed to obtain two cell suspensions, consisting respectively of myogenic cells (CD56^+^) and non-myogenic mesenchymal cells (CD56^−^). The CD56^−^ fraction was subsequently cultured in CYTO-GROW medium in untreated flasks. The immunophenotype of the CD56^−^ cell population of four biological replicates for each experimental group was further characterised by analysing the expression of mesenchymal, hematopoietic and endothelial surface markers by flow cytometry using the antibodies listed in Supplementary Table S1. Cell suspensions were acquired on a Cytoflex LX flow cytometer (Beckman Coulter) after staining at room temperature (RT) for 15 min, in the presence of a live/dead discrimination dye.

### Adipogenic induction and Oil Red O staining

Non-myogenic mesenchymal cells were plated at a density of 5 × 10^3^ cells/cm^2^ and grown until confluence for 72 h, at which point the proliferative medium was replaced by an inductive adipogenic medium [Dulbecco’s modified Eagle medium (DMEM) with sodium pyruvate (Aurogene) supplemented with 10% of fetal bovine serum (FBS, GIBCO by Thermo Fisher Scientific, Waltham, MA, USA), 0.5 mM 3-isobutyl-1-methylxanthine (Sigma-Aldrich, St. Louis, Missouri, Stati Uniti), 0.25 μM dexamethasone (Sigma-Aldrich), 10 µg/ml insulin (Sigma-Aldrich), 1% l-glutamine (Euroclone, Milan, Italy), 1% antibiotics (Aurogene)]. On the third day, the adipogenic medium was replaced with an adipogenic differentiation maintenance medium (DMEM with sodium pyruvate supplemented with 10% FBS, 10 µg/ml insulin, 1% l-glutamine and 1% antibiotics), that was changed twice a week. The differentiative potential was assessed after 7 and 14 days. Cells between the 3rd and 5th culture passage were used. The experiment was replicated in all the primary cultures established as biological replicates and also, as needed, as technical replicates for specific samples. Cells fixed in 4% (w/v) formalin were stained with an Oil Red O solution (5 mg/ml of Oil Red O in 2-propanol deionized water). At least six not-overlapping images for sample were acquired with optical microscope Primovert (Zeiss, Jena, Germany) at ×10 magnification, using the ZEN 2.5 Blu edition software (Zeiss). The images obtained were analysed using the ImageJ software to quantify the area occupied by lipids. Each image was converted to a grayscale image (Image, Type, RGB stack) and, by using the ImageJ Brightness/Contrast tool, the lipid droplets were highlighted as white droplets on a black background. White area measurement corresponded to the lipid droplet area per field.

### RNA isolation and gene expression analysis

Total RNA was isolated from frozen muscle specimens and from cell cultures using Trizol reagent (Thermo Fisher Scientific), following manufacturer’s procedures. RNA was subsequently purified using GeneAll Riboclear™ (Plus) kit, including a DNase I digestion step for the complete removal of genomic DNA. RNA quantity was assessed using the Nanodrop One spectrophotometer (Thermo Fischer Scientific). Up to 500 ng of total RNA were reverse transcribed using the GoScript™ Reverse Kit (Promega, San Luis Obispo, CA, USA) and the expression of selected mesenchymal, adipogenic and fibrogenic genes was quantified by real-time quantitative PCR (qRT-PCR) with Syber Green master mix (GoTaq qPCR Master Mix Kit, Promega), and custom sequence-specific oligonucleotide primer pairs (listed in Supplementary Table S2). The relative expression levels were normalised to *glyceraldehyde-3-phosphate dehydrogenase* (*GAPDH*) or *actin beta* (*ACTB*) levels and quantified according to the 2^−∆∆Ct^ method [27].

### Immunofluorescence and histological analyses

Seven µm thick sections of fresh frozen muscle specimens were obtained with cryostat at 25 °C. For immunofluorescence analysis, tissue sections were incubated in pre-cooled acetone (Carlo Erba reagents, Milan, Italy) at −20 °C for 6 min and subsequently in PBS with 5% Normal Donkey serum (Jackson ImmunoResearch Europe Ltd, Ely, UK) and 1% bovine serum albumin (BSA, Sigma-Aldrich) for 1 hour at RT to block nonspecific sites. Sections were incubated with the following primary antibodies in PBS with 1% of BSA: anti-laminin (1:400, #L9393, Sigma-Aldrich), anti-CD201 (1:100, #AF2245, R&D systems, Minneapolis, MN, USA) anti-PDGFRα (1:500, #3174, Cell Signalling Technology, Danvers, MA, USA) and anti-PDGFRα (1:100, #AF-307-NA, R&D systems). The following secondary antibodies were used and incubated for 45 min at RT in PBS with 1% of BSA: anti-Rabbit CY3 (1:100, #711-165-152, Jackson ImmunoResearch) and anti-Goat Alexa Fluor 488 (1:100, #305-545-045, Jackson ImmunoResearch). All incubations were followed by 3 washes in PBS added with 0.1% Tween 20 (Sigma-Aldrich). Cell nuclei were counterstained with a mounting medium solution with DAPI (#H-1200, Vector Laboratories Ltd, Peterborough, UK). Two non-consecutive tissue sections from each sample were processed for each combination of antibodies, and consecutive tissue sections were analysed for the different antibodies. At least five non-overlapping images representing each slide were acquired with the Axiophot fluorescence microscope (Zeiss) at ×20 magnification using the ZEN 2.5 Blu edition software. For the colocalization analysis of CD201^+^ and PDGFRA^+^ non-myogenic mesenchymal cells, images were acquired at Nikon confocal laser scanning microscopy system (A1MP+, Nikon, Amsterdam, Netherlands).

Muscle fibrosis were measured using Picrosirius red staining. Muscle sections fixed in paraformaldehyde 4%, were incubated with a 0.1% Picrosirius red solution (Direct Red 80 #365548, Picric Acid Solution #P6744, Sigma-Aldrich). Samples were washed in distilled water with 0.5% acetic acid (J.T.Baker by Thermo Fisher Scientific), dehydrated in ethanol (VWR Chemical, Radnor, PA, USA) and fixed in xylene (VWR Chemical). Non-overlapping images were acquired at ×20 magnification with the Olympus BH-2 microscope (Olympus, Tokyo, Japan). Collagen deposition was quantified by the ImageJ software: each image was uploaded and converted to a grayscale image (Image, Type and RGB stack) and, using the ImageJ Threshold tool, modified to display the fibrotic areas and the percentage of fibrosis was measured.

### Statistical analysis and multivariate data analysis

All statistical analyses were performed using GraphPad Prism software (San Diego, CA, USA). Comparisons between the different experimental groups were alternatively assessed using the one-way analysis of variance (ANOVA) followed by Tukey’s multiple comparison test or non-parametric Kruskal–Wallis comparison test, or by non-parametric Mann–Whitney *t* test. Linear correlation analyses were instead performed using the Pearson’s correlation test. The level of significance was set at *p* < 0.05. Hierarchical clustering and principal component analysis (PCA) were done with ClustVis, a web tool written in R [28]. Original values were ln(x)-transformed and pareto scaling was applied to rows; Singular Vector Decomposition was used to calculate principal components.

## Results

### Immunophenotypic characterisation of human non-myogenic mesenchymal cells from FSHD and control muscles

**Fig. 1:**
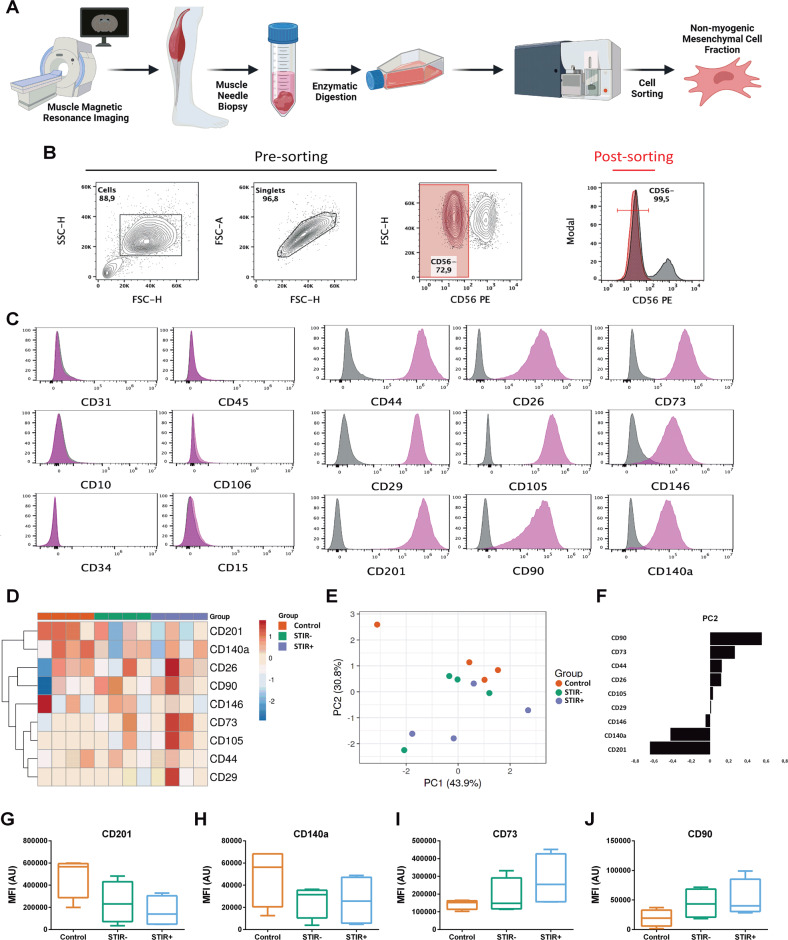
Isolation and characterisation of non-myogenic mesenchymal cells. **A** Schematic representation of non-myogenic mesenchymal cell isolation protocol (panel created with Biorender.com). **B** Gating strategy for non-myogenic mesenchymal cell sorting from the CD56^−^ cell fraction. **C** Representative histograms of specific markers in the CD56^−^ cell fraction. **D** Heatmap showing the relative expression levels of positive markers. Original values are ln(x)-transformed. Rows are centred and scaled. Rows are clustered using Euclidean distance and Ward linkage. 9 rows, 13 columns. **E** PCA analysis by singular value decomposition of the same dataset showed in **D**. *X* and *Y* axis show principal component 1 (PC1) and principal component 2 (PC2). The percentage of the total variance explained by each PC is indicated. **F** Loadings (i.e. contributions) of each variable to PC2. **G**–**J** Relative expression, expressed as median fluorescence intensity (MFI) of CD201, CD140a, CD73 and CD90.

Muscle specimens were collected through needle muscle biopsies after MRI examination. The adherent fraction of cells, obtained after the enzymatic digestion of four control, four FSHD STIR− and five FSHD STIR+ muscles, was successfully expanded in vitro, as previously reported [6, 29–31] (Fig. 1A). The four FSHD STIR− muscles used for this experiment were also negative in T1, whereas all the five FSHD STIR+ muscles showed different degrees of adipose tissue infiltration, assessed by enhanced signal on T1w sequences. Non-myogenic mesenchymal cells were then sorted by flow cytometry as the CD56^−^ cell fraction (Fig. 1B). The proportions of CD56^−^ and CD56^+^ cells were sample dependent and the mean percentage of CD56^−^ cells obtained after cell sorting was comparable across the three different conditions analysed (mean percentage of CD56^−^ fraction ± SD: 93.5 ± 6.4 in controls, 85.3 ± 15.2 in FSHD STIR−, 84.2 ± 17.4 in FSHD STIR+). To better characterise the immunophenotype of cultured CD56^−^ cells and to compare it with patient-derived and control samples, the expression of a panel of specific markers was evaluated by flow cytometry. CD56^−^ cells were positive for specific mesenchymal markers, such as CD44, CD26, CD73, CD29, CD105, CD146, CD201, CD90 and CD140a (PDGFRA) (Fig. 1C), in keeping with previous reports on muscle-resident non-myogenic mesenchymal cells [29, 31–33]. CD56^−^ cells did not express typical hematopoietic and endothelial markers CD31, CD45, CD10, CD106, CD34 and CD15 (Fig. 1C). The relative expression of different markers was examined by visualising median fluorescence intensities in a heatmap (Fig. 1D) and by PCA (Fig. 1E) to evaluate possible differences between non-myogenic mesenchymal cells isolated from control, FSHD STIR− and FSHD STIR+ muscles. Our analysis showed that cells isolated from the muscles of FSHD patients were phenotypically different from control cells (Fig. 1E) based on the distinct expression of CD90, CD73, CD140a and CD201 (Fig. 1F). Indeed, considering the expression of single markers, there was a trend, even if not significant, toward decreased CD201 and CD140a (Fig. 1G, H) and increased CD73 and CD90 expression in cells isolated from FSHD STIR+ muscles compared with control cells (Fig. 1I, J).

### Patient-derived non-myogenic mesenchymal cells showed an impaired adipogenic differentiation

**Fig. 2:**
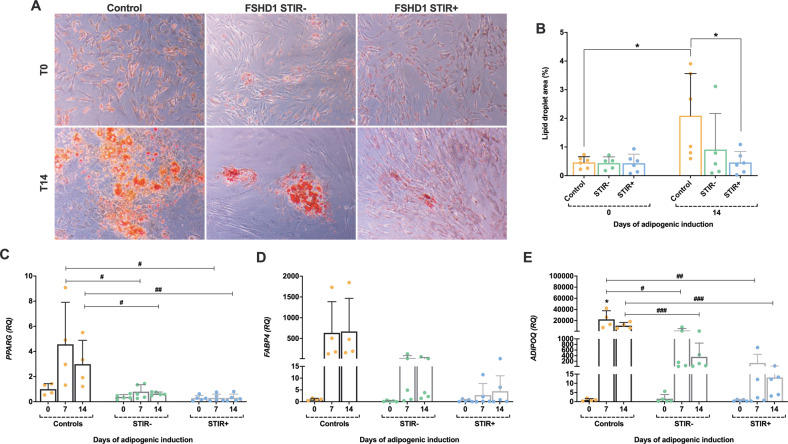
Adipogenic induction of non-myogenic mesenchymal cells. **A** Representative picture of non-myogenic mesenchymal cells treated with adipogenic medium and analysed for intracellular lipid droplet formation with oil red O staining at day 0 (T0, when the proliferative medium was replaced with the adipogenic medium) and 14 days (T14) after the start of differentiation (×10 magnification). **B** Lipid droplets were quantified using ImageJ software and the results are reported in the graph bar as mean ± SD. *P* values were assessed by ordinary one-way ANOVA followed by Tukey’s multiple comparisons test; *p < 0.05. **C**–**E** Relative expression analysis of *PPARG*, *FABP4,* and *ADIPOQ* evaluated by RT-qPCR, at day 0 and after 7 and 14 days of adipogenic induction. The relative quantity (RQ) was calculated according to the 2^−ΔΔCt^ method normalised to *ACTB* expression, and the day 0 value of control mesenchymal cells was set as a reference for each experimental group. Data are presented as mean ± SD. *P* values were assessed by ordinary one-way ANOVA followed by Tukey’s multiple comparisons test; *p < 0.05 versus control T0; ^#^*p* < 0.05, ^##^*p* < 0.01; ^###^*p* < 0.001.

Non-myogenic mesenchymal cells were induced to differentiate towards the adipogenic lineage for 14 days and afterwards the acquired phenotype was studied and compared in cells isolated from control and FSHD muscles. In control cells there was a significant increase in the percentage of area occupied by lipids 14 days after starting the induction compared with time 0 (T0) (Fig. 2A, B). Conversely, mesenchymal cells isolated from FSHD STIR+ muscles did not respond to the inductive stimulus: the percentage of area filled with lipid droplets did not increase after 14 days of differentiation and was significantly different from that of control cells (Fig. 2A, B). In addition, our data highlighted that the behaviour of non-myogenic mesenchymal cells derived from “unaffected” FSHD muscles was heterogenous and some muscles featured mesenchymal cells preserving the capacity to undergo adipogenic differentiation (Fig. 2A, B).

The relative expression of adipogenic specific markers [namely *peroxisome proliferator-activated receptor gamma* (*PPARG*), *fatty acid binding protein 4* (*FABP4*), *adiponectin, C1Q and collagen domain containing* (*ADIPOQ*) genes] was also assessed at T0, and after 7 (T7) and 14 (T14) days of induction. *FABP4* and *ADIPOQ* transcript levels were higher at T7 and T14 when compared to T0 cells, in both control and in FSHD muscles (Fig. 2D, E). *PPARG* expression increased only in control cultures during the adipogenic induction (Fig. 2C). Nonetheless, by comparing control- and patient-derived cell expression at each time-point, non-myogenic mesenchymal cells isolated from FSHD STIR− and FSHD STIR+ muscles displayed lower levels of the markers analysed (Fig. 2C–E). In particular, the expression levels of both *PPARG* and *ADIPOQ* were significantly downregulated in FSHD patient-derived cells compared with control cells at T7 and T14 (Fig. 2C, E).

We also evaluated the expression of *protein C receptor* (*PROCR*, alias *CD201*), *platelet-derived growth factor receptor alpha* (*PDGFRA*) and *Thy1 cell surface antigen* (*THY1*, alias *CD90*) genes during differentiation. Our results showed that, while the mean relative expression of *CD201* and *PDGFRA* did not change in any experimental group analysed (Supplementary Fig. S1A, B), there was a significant upregulation of *CD90* in non-myogenic mesenchymal cells isolated from control and FSHD STIR+ muscles after 14 days of adipogenic induction compared with T0 (Supplementary Fig. S1C). However, by comparing patient-derived and control cells at the different time-points, a significant downregulation of *CD201* levels could be observed in FSHD STIR+ cells compared with controls after 14 days of adipogenic induction (Supplementary Fig. S1A). Also, *PDGFRA* transcript levels were significantly downregulated in FSHD STIR+ cells compared with control cells after 7 days of induction; past this time-point, the differential expression was not statistically significant (Supplementary Fig. S1B). *CD90* mean levels were instead comparable between control and patient-derived cultures at the different time-points tested, although the expression of this gene was higher in three out of five FSHD STIR− cell cultures than in control and FSHD STIR+ cells (Supplementary Fig. S1C).

### Expansion of CD201^+^ and PDGFRA^+^ non-myogenic mesenchymal cells in FSHD STIR+ muscles

Given the results obtained in our in vitro model, we also evaluated non-myogenic mesenchymal cell localisation and distribution in a larger cohort of control and FSHD skeletal muscle sections using CD201 and PDGFRA as markers [29, 32]. Stains with both CD201 and PDGFRA were performed since we observed, by means of preliminary colocalization analysis, that the two markers occasionally did not label the same cells (Supplementary Fig. S2). CD201^+^ and PDGFRA^+^ cells localised between the basal lamina of adjacent muscle fibres, and were also observed around blood vessels.

**Fig. 3:**
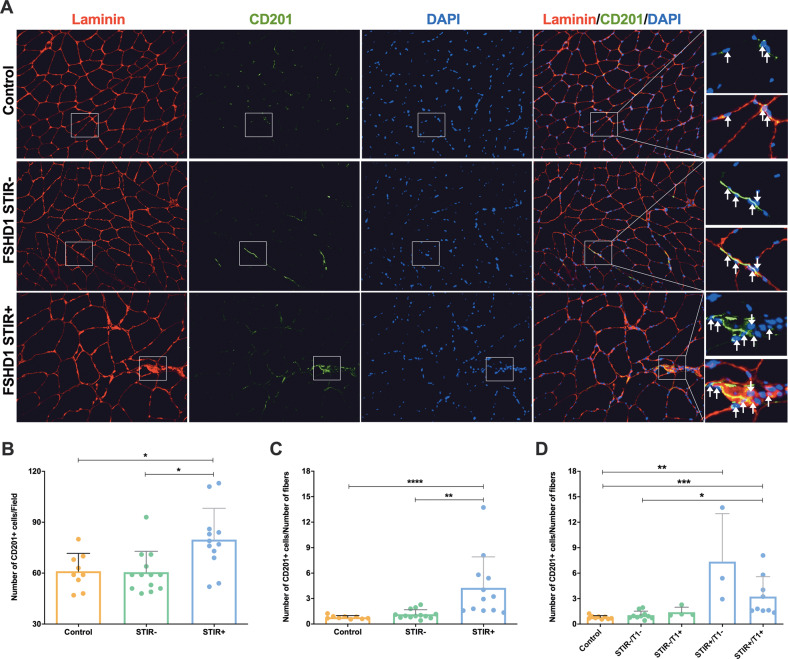
Expansion of CD201^+^ non-myogenic mesenchymal cells in FSHD muscles. **A** Immunofluorescence staining for laminin (red), non-myogenic mesenchymal cells with CD201 (green), and nuclei with DAPI (blue). ×20 magnification. On the right, two enlarged representative areas showing CD201^+^ cells for each experimental group are shown. **B** The number of CD201^+^ cells/field was reported as mean ± SD for each group. The ratio between the number of CD201^+^ cells and the number of myofibers per field was assessed considering only the MRI parameter on STIR sequences (**C**) or also the T1 MRI value (**D**). The results were reported as mean ± SD. For all the analysis *p* values were assessed by one-way ANOVA followed by Kruskal–Wallis multiple comparisons post hoc test; *p < 0.05, **p < 0.01, ***p < 0.001, ****p < 0.0001.

The number of CD201^+^ cells per field was significantly higher in FSHD STIR+ compared with FSHD STIR− and control ones (Fig. 3A, B). To also consider the characteristic presence of hypertrophic fibres in FSHD STIR+ muscles, compared with both control and FSHD STIR− muscles (Fig. 3A), we also analysed and compared the number of non-myogenic mesenchymal cells per fibre in each field. The ratio between the number of CD201^+^ cells and the number of fibres per field was significantly higher in FSHD STIR+ muscles than in both FSHD STIR− and control muscles (Fig. 3A, C). To take into account the presence/absence of fatty replacement in single muscles, the signal on T1w sequences were also considered, where T1− signal means no adipose tissue presence, whereas T1+ stands for muscle fatty substitution. Clustering FSHD muscles as STIR−/T1−, STIR−/T1+, STIR+/T1− and STIR+ T1+ according to the MRI findings, we observed that the number of CD201^+^ cells per number of fibres significantly increased in both STIR+/T1− and STIR+/T1+ muscles compared with controls, and in STIR+/T1+ compared with STIR−/T1− (Fig. 3D).

**Fig. 4:**
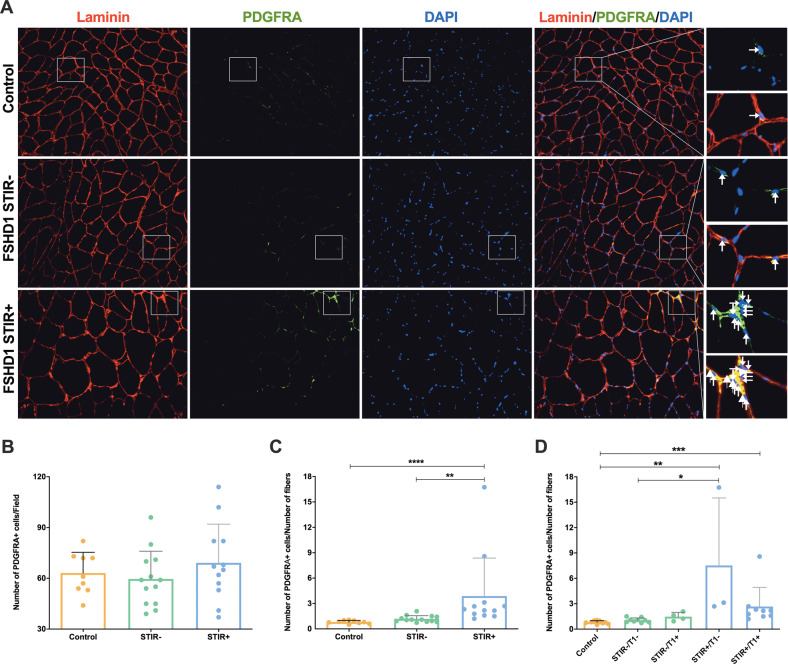
Expansion of PDGFRA+ non-myogenic mesenchymal cells in FSHD muscles. **A** Immunofluorescence staining for laminin (red), non-myogenic mesenchymal cells with PDGFRA (green) and nuclei with DAPI (blue). ×20 magnification. On the right, there are two enlarged representative areas showing PDGFRA^+^ cells for each experimental group. **B** The number of PDGFRA^+^ cells/field was reported as mean ± SD for each group. The ratio between the number of PDGFRA^+^ cells and the number of myofibers per field was assessed considering only the MRI parameter on STIR sequences (**C**) or also the T1 MRI value (**D**). The results were reported as mean ± SD. For all the analysis p-values were assessed by one-way ANOVA followed by Kruskal–Wallis multiple comparisons post hoc test; *p < 0.05, **p < 0.01, ***p < 0.001, ****p < 0.0001.

The same analysis using PDGFRA as marker of non-myogenic mesenchymal cells showed that, while there were no differences in the number of PDGFRA^+^ cells between the different muscles analysed (Fig. 4A, B), the ratio between the number of PDGFRA^+^ cells and the number of fibres per field was significantly increased in FSHD STIR+ muscles compared with FSHD STIR− and control muscles (Fig. 4A, C). As for CD201, a significant difference was maintained for both FSHD STIR+ groups separately considered (STIR+/T1− and STIR+/T1+ muscles) compared with control muscles (Fig. 4D). Of note, the number of PDGFRA^+^ cells per number of fibres was significantly higher in FSHD STIR+/T1− compared to FSHD STIR−/T1− muscles (Fig. 4D).

### Correlation between non-myogenic mesenchymal cell expansion, fibrosis, and fat accumulation in FSHD muscles

**Fig. 5:**
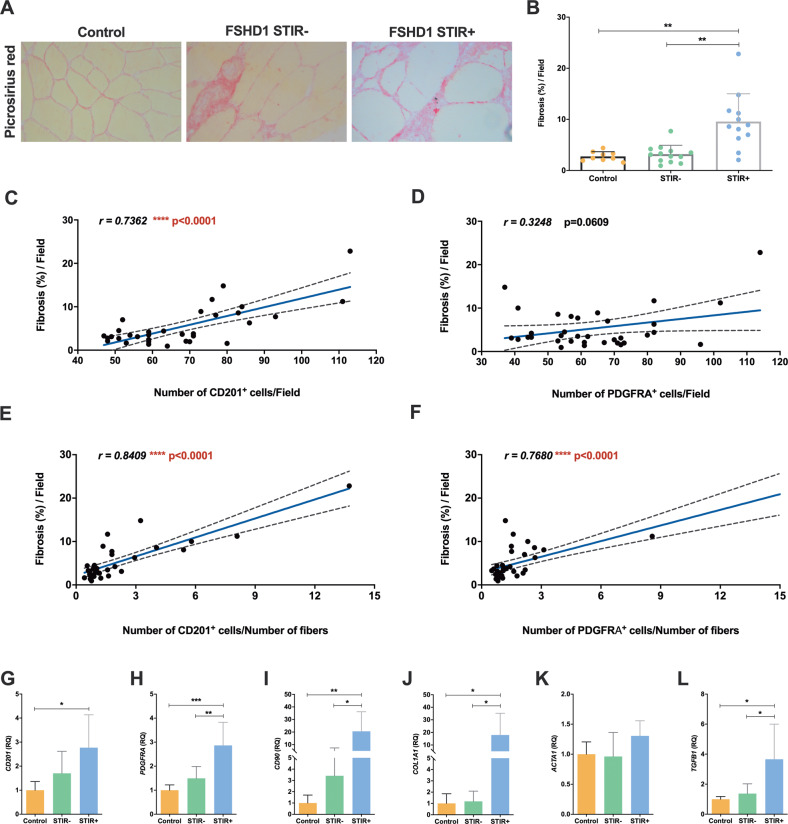
Correlation between mesenchymal cell expansion and fibrosis in FSHD muscles. **A** Picrosirius red staining of skeletal muscle sections for fibrosis. ×20 magnification. **B** Percentage of fibrosis per field reported as mean ± SD. *P* values were assessed by one-way ANOVA followed by Kruskal–Wallis multiple comparisons post hoc test; **p < 0.01. Pearson correlation between the number of CD201^+^ cells per field (**C**), the number of PDGFRA^+^ cells per field (**D**), the number of CD201^+^ cells/number of myofibers per field (**E**) or the ratio of PDGFRA^+^ cells/number of myofibers per field **(F)** and fibrosis. The relative Pearson correlation coefficient (*r* value) and *p* value (*p*) are reported. **G**–**L** Relative expression (RQ) of *CD201*, *PDGFRA*, *CD90*, *COL1A1*, *ACTA1* and *TGFB1* mRNA, normalised to *GAPDH* levels and calculated according to the 2^-ΔΔCt^ method, setting the value of controls as reference. Data are represented as mean ± SD and *p* values were assessed by ordinary one-way ANOVA followed by Tukey’s multiple comparisons test; *p < 0.05, **p < 0.01, ***p < 0.001. Control muscles: *n* = 6, FSHD STIR− muscles: *n* = 5, FSHD STIR+ muscles: *n* = 5.

We then correlated the expansion of CD201^+^ and PDGFRA^+^ non-myogenic mesenchymal cells with the deposition of fibrous and adipose tissue in FSHD muscles. Picrosirius red staining revealed a significant difference in fibrosis between control, FSHD STIR− and FSHD STIR+ muscles (Fig. 5A, B). Interestingly, we observed a significant positive correlation between the percentage of fibrosis observed in each muscle section and the number of CD201^+^ cells (Fig. 5C). The correlation between the extent of fibrosis and the number of PDGFRA^+^ cells was not significant (Fig. 5D). The percentage of fibrosis instead positively correlated with the ratio between the number of both CD201^+^ and PDGFRA^+^ cells and the fibre number (Fig. 5E, F). On the contrary, all the measure of the expansion of CD201^+^ and PDGFRA^+^ non-myogenic mesenchymal cells did not correlate with the T1 score, accounting for different degrees of adipose tissue infiltration of each muscle globally evaluated by MRI in FSHD patients (Supplementary Fig. S3A–D). To confirm these data, we also quantified the whole-muscle expression of different mesenchymal, fibrotic and adipogenic markers in control, FSHD STIR− and FSHD STIR+ muscles. The total expression of *CD201*, *PDGFRA*, *CD90*, as well as of *collagen type I alpha 1 chain* (*COL1A1*) and *transforming growth factor beta 1* (*TGFB1*), 2 key fibrotic genes, was significantly increased in FSHD STIR+ muscles compared with control ones (Fig. 5G–J, L). Of note, *PDGFRA*, *CD90, COL1A1* and *TGFB1* transcript levels resulted upregulated in FSHD STIR+ muscles also compared with FSHD STIR− muscles (Fig. 5H–J, L). Instead, the expression levels of *actin alpha 1, skeletal muscle* (*ACTA1*, Fig. 5K) and of the adipogenic markers *PPARG*, *FABP4* and *ADIPOQ* (Supplementary Fig. S3E–G) did not vary between control and FSHD patient muscles.

**Fig. 6:**
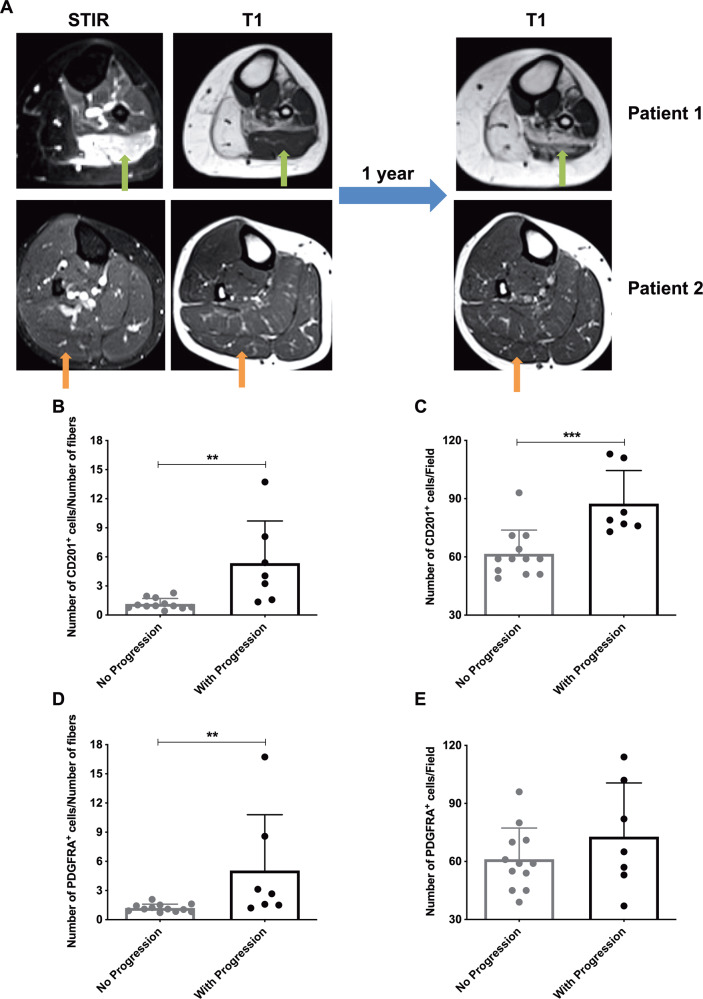
Correlation of non-myogenic mesenchymal cell expansion with progression of fatty degeneration. **A** Patient 1 (FSHD#25): left gastrocnemius lateralis (green arrows) appears hyperintense on STIR sequence (STIR+) and normal on T1w sequence (no fat substitution). After 1-year follow-up T1w sequence showed partial fat substitution. Patient 2 (FSHD#8): right gastrocnemius lateralis (orange arrows) appears normal on STIR sequence (STIR−) and normal in T1w sequence (no fat substitution). After 1-year follow-up T1w sequence showed no fat substitution. Ratio of CD201^+^ cells/number of myofibers/field (**B**) and number of CD201^+^ cells/field (**C**), in muscles that did not worsen and that had a worsened on T1w sequences at follow-up at MRI, respectively. Ratio of PDGFRA^+^ cells/number of myofibers/field (**D**) and number of PDGFRA^+^ cells/field (**E**), in muscles that did not worsen and that worsened on T1w sequences at follow-up at MRI, respectively. For all the graphs, results are presented as mean ± SD and p-values were assessed by one-way ANOVA followed by Kruskal–Wallis multiple comparisons post hoc test; **p < 0.01, ***p < 0.001.

Finally, given that for 19 patients it was possible to record disease progression at the single-muscle level by analysing the worsening of T1 signal at the follow-up with MRI after 1 year (Fig. 6A), we studied the correlation of non-myogenic mesenchymal cell expansion with this parameter. The ratio between the number of CD201^+^ cells/number of fibres, as well as the number of CD201^+^ cells/field, was significantly higher in FSHD muscles that showed increased fatty degeneration at follow-up (Fig. 6B, C). Also, the ratio between the number of PDGFRA^+^ cells/number of fibres was significantly higher in FSHD muscles that showed progression on fat degeneration at follow-up (Fig. 6D), whereas the same trend could not be observed considering the number of PDGFRA^+^ cells per field (Fig. 6E).

## Discussion

In the FSHD research field, several efforts are devoted to clarifying the pathogenetic mechanisms caused by the inappropriate expression of *DUX4* in patients’ muscles by evaluating the effects of DUX4 molecular signature on myogenic cells [34–36]. We wondered whether other non-myogenic cell subpopulations could also affect degenerative processes within the skeletal muscle stem cell niche, thus contributing to FSHD pathogenesis. In this study, we focused on the characterisation of non-myogenic mesenchymal cells as potential contributors to FSHD pathophysiology in order to suggest alternative degenerative mechanisms or worsening factors in patients. Thanks to the information provided by MRI at the single-muscle level, we investigated the events occurring in the stem cell niche of FSHD muscles in which the signs of disease are evident (STIR+ muscles), compared with both apparently normal FSHD (STIR−) and control muscles.

Our results demonstrate for the first time that FSHD patients’ muscles are characterised by an increased number of both CD201^+^ and PDGFRA^+^ non-myogenic mesenchymal cells, and that this change is prominent in muscles displaying signs of early damage. Of note, an increased number of non-myogenic mesenchymal cells is strictly associated with the decreased number of myofibers and with their characteristic hypertrophy, as a hallmark of FSHD-affected muscles [37]. This is demonstrated in our study by the increased ratio between the number of CD201^+^ and PDGFRA^+^ cells and the number of fibres per field in FSHD STIR+ muscles. This is more evident considering PDGFRA expression. The data obtained in this work also suggest that the accumulation of non-myogenic mesenchymal cells positively correlates with muscle fibrosis and with signs of muscle inflammation associated to a hyperintensity signal on STIR MRI sequences, rather than a parameter associated with fat accumulation on T1w sequences. Interestingly, considering fat degeneration at the single-muscle level assessed by MRI within 1 year after biopsy, the expansion of CD201^+^ and PDGFRA^+^ non-myogenic mesenchymal cells is higher in FSHD muscles that progress more rapidly in T1 signal than in those that do not progress. Thus, the accumulation of CD201- and PDGFRA-expressing cells could be related to an early stage of muscle degeneration, observed as STIR positivity in patients, which is particularly associated with muscle inflammation. Thereafter, fibrosis could hasten a subsequent degeneration that includes aberrant fat tissue accumulation in the FSHD skeletal muscle, as revealed by the longitudinal analysis.

Muscle niche homoeostasis is regulated by a complex signalling network of bioactive factors in the local environment, including cytokines, growth factors and adhesion molecules of the extracellular matrix, secreted by several cell types [7]. The dysregulation of this paracrine crosstalk, mainly associated with persistent inflammation in patients’ muscles [8, 14], might reasonably have an active role in the degenerative processes observed in FSHD patients. In particular, the inflammatory environment might affect the differentiative potential of non-myogenic mesenchymal progenitors leading to an accumulation of collagen deposits in patient STIR+ muscles and the fibrosis could in turn promote fat deposition. It has been earlier suggested that fibrosis might accompany fat deposition [38]. In this study, we hypothesise that the early phases of muscle degeneration could stimulate the adipogenic programme in the remaining pool of non-myogenic mesenchymal progenitors or in a specific subpopulation of them within affected patients’ muscles.

We also show that non-myogenic mesenchymal cells isolated from FSHD STIR+ muscles express a distinct pattern of surface markers, which might reflect different functional state of cells residing in patients’ muscles in comparison with cells isolated from healthy muscles. Specifically, non-myogenic mesenchymal cells isolated from FSHD STIR+ muscles display a decreased expression of CD201 that was previously found downregulated in differentiated adipocytes [32], as well as an increased expression of CD90, whose upregulation promotes fibrogenesis [9]. In addition, mesenchymal cells isolated from both STIR− and STIR+ FSHD muscles are not able to respond to stimuli and differentiate toward mature adipocytes in vitro. Even though this observation might, at first glance, looks incoherent with data obtained from muscle tissue analyses, we believe that the cells we isolated and analysed in vitro represent only a proportion of the heterogeneous population expressing CD201 and PDGFRA in the muscle stem cell niche that can be observed in vivo. First, culture expanded cells certainly cannot show the more differentiated status that is not maintained with culture passages. Then, the cell population isolated from FSHD patients’ muscles is particularly enriched in fibroblasts and hence do not reflect the in vivo adipogenic differentiation potential of non-myogenic mesenchymal progenitors. Furthermore, the complexity of the niche, including all signals deriving from the different cell types that populate the muscle, is only partially reproduced in vitro and the culture conditions could differentially influence the responsiveness of non-myogenic mesenchymal cells to the extracellular cues. Finally, the selected markers can also label other cells within the muscle stem cell niche as immune cells or other precursors cells [39], thus further analysis would enable better defining which cells are more precisely involved in the pathogenesis of FSHD.

Analyses of histological sections from STIR− FSHD muscles (i.e., with no objective sign of disease activity) show that non-myogenic mesenchymal cells are not expanded. Also, cells isolated from STIR− muscles appear to have an intermediate behaviour between control cells and STIR+ muscle-derived cells. We, therefore, hypothesise that non-myogenic mesenchymal cells might receive altered stimuli, resulting from the genetic defects underlying the disease, also in these muscles. Nonetheless, in STIR− muscles, protective mechanisms might be activated, including specific intercellular paracrine signalling mediated by other cell types within the stem cell niche, being able to promote the resolution of the steady-state condition of non-myogenic mesenchymal cells.

In the last years, several works have aimed at clarifying the implication of non-myogenic mesenchymal cells in the pathogenesis of neuromuscular diseases, for instance Duchenne muscular dystrophy or dysferlinopathy among others [9, 40–43]. However, non-myogenic mesenchymal cells’ involvement in FSHD pathophysiology has been so far investigated exclusively in a murine model. Our results in patients are in agreement with recent findings obtained in the iDUX4pA mouse model by Bosnakovski and colleagues [17–19], who demonstrated a long-lasting increase of Pdgfrα^+^ cells, that followed the inducible expression of DUX4, and reflected a pro-fibrotic and anti-regenerative state in the skeletal muscle [17–19].

Taken together, this evidence supports the view that FSHD muscles undergo degeneration through different disease stages marked by clearly different molecular changes that also differently affect the intramuscular milieu, and have a conditioning effect on different cell types within the skeletal muscle stem cell niche.

**Fig. 7:**
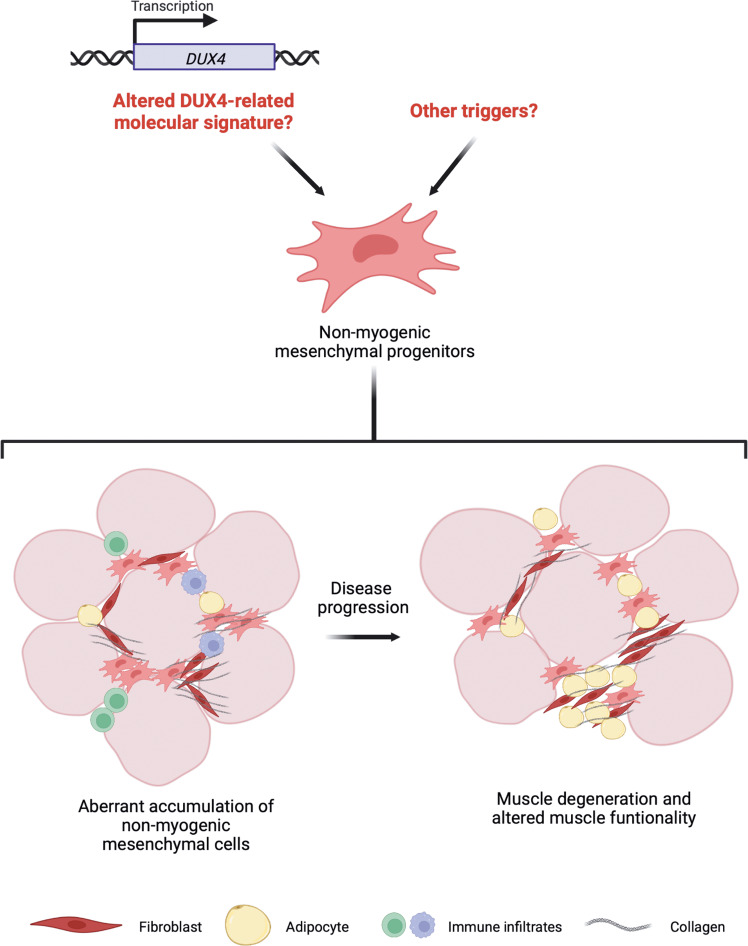
Altered behaviour of non-myogenic mesenchymal progenitors in FSHD patients’ muscles. An excessive accumulation, as well as an aberrant differentiation of non-myogenic mesenchymal progenitors can be observed in FSHD patients’ muscles in which the active phase of the disease is identifiable by muscle MRI. Although the causes of non-myogenic mesenchymal progenitors’ dysregulation are not clear, this process might lead to muscle wasting of selected muscles in FSHD patients. (Created with BioRender.com).

However, it remains to be clarified what triggers the altered behaviour of non-myogenic mesenchymal cells in FSHD patients and what is the temporal order of events (Fig. 7). Further studies are necessary to better clarify the possibility of a direct alteration of DUX4-related molecular signature in these cells. Another option could be that the altered *DUX4* gene expression in degenerating fibres and the inflammatory processes that characterise STIR+ muscles may create a detrimental environment able to affect the physiology of non-myogenic mesenchymal cells, which in turn drive the terminal stages of muscle degeneration in patients. In conclusion, our results pave the way towards understanding the contribution of non-myogenic mesenchymal cells to muscle degeneration in FSHD patients and could provide essential clues to identify novel therapeutic targets that might contrast the evolution of the disease and sustain tissue regeneration in patients.

## Supplementary information

Additional supplementary material file

Supplementary Table S1

Supplementary Table S2

Supplementary Figure S1

Supplementary Figure S2

Supplementary Figure S3

Checklist

## Data Availability

All the results obtained in this study are presented in the article. No new primary datasets to be deposited have been generated. The data that support the findings of this study are available from the corresponding author upon reasonable request.
