# Supplementary material for: Non-myogenic mesenchymal cells contribute to muscle degeneration in facioscapulohumeral muscular dystrophy patients
Source: Cell Death Dis. 2022 Sep 16;13(9):793. doi: 10.1038/s41419-022-05233-6 (PMC9481542; doi:10.1038/s41419-022-05233-6)
Supplement: Supplementary file 1 — Additional supplementary material file [file 41419_2022_5233_MOESM1_ESM.docx]

**Supplementary information**

**Supplementary Table S1**

**Supplementary Table S2**

**Supplementary Fig. S1. Relative expression of *CD201*, *PDGFRA* and *CD90*** **during adipogenic induction *in vitro*.** The relative quantity (RQ) of the different transcripts was calculated according to the 2^-ΔΔCt^ method and normalized to *ACTB* expression, alternatively setting the day 0 value of control mesenchymal cells as reference for each experimental group and each time-point analysed. Data are presented as mean ± SD, and p-values were assessed by ordinary one-way ANOVA followed by Tukey’s multiple comparisons test; **p<0.01 versus control T0; #p<0.05, ##p<0.01.

**Supplementary Fig. S2. Colocalization of CD201^+^ and PDGFRA^+^ mesenchymal cells in muscle sections.** Immunofluorescence staining for non-myogenic mesenchymal cells with CD201 (green), PDGFRA (red) and nuclei with DAPI (blue). 10X magnification. On the right, from top to bottom and for control, FSHD1 STIR- and FSHD1 STIR+ muscles respectively, there are three enlarged representative areas showing cells with colocalization CD201^+^ PDGFRA^+^ (merged signal), only CD201^+^ cells (green) and PGFRA^+^ cells (red).

**Supplementary Fig. S3.** **Correlation of non-myogenic mesenchymal cell expansion with adipogenesis in FSHD muscles.** Pearson correlation between the number of CD201^+^ cells per field **(A)**, the number of PDGFRA^+^ cells per field **(B)**, the ratio of CD201^+^ cells/number of myofibers per field **(C)** or the ratio of PDGFRA^+^ cells per field **(D)** and T1 MRI score. **E-G** Relative expression (RQ) of *PPARG*, *FABP4* and *ADIPOQ* mRNA, normalized to *GAPDH* levels and calculated according to the 2^-ΔΔCt^ method, setting the value of controls as reference. Data are represented as mean ± SD. Control muscles: n=6, FSHD STIR- muscles: n=5, FSHD STIR+ muscles: n=5.
