# Supplementary figures and images for: Non-myogenic mesenchymal cells contribute to muscle degeneration in facioscapulohumeral muscular dystrophy patients
Source: Cell Death Dis. 2022 Sep 16;13(9):793. doi: 10.1038/s41419-022-05233-6 (PMC9481542; doi:10.1038/s41419-022-05233-6)

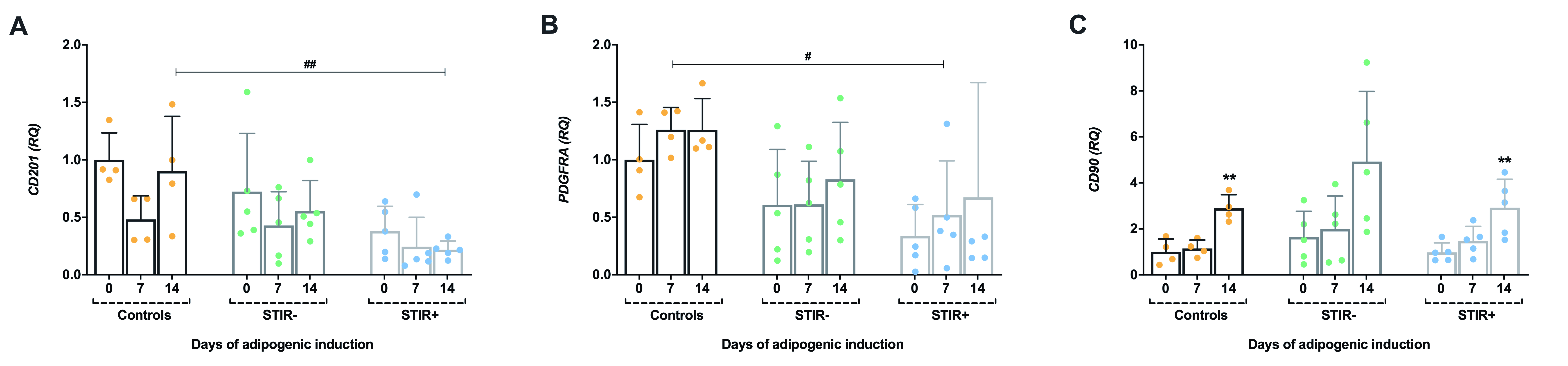

Supplement: Supplementary file 4 — Supplementary Figure S1 [file 41419_2022_5233_MOESM4_ESM.tif]

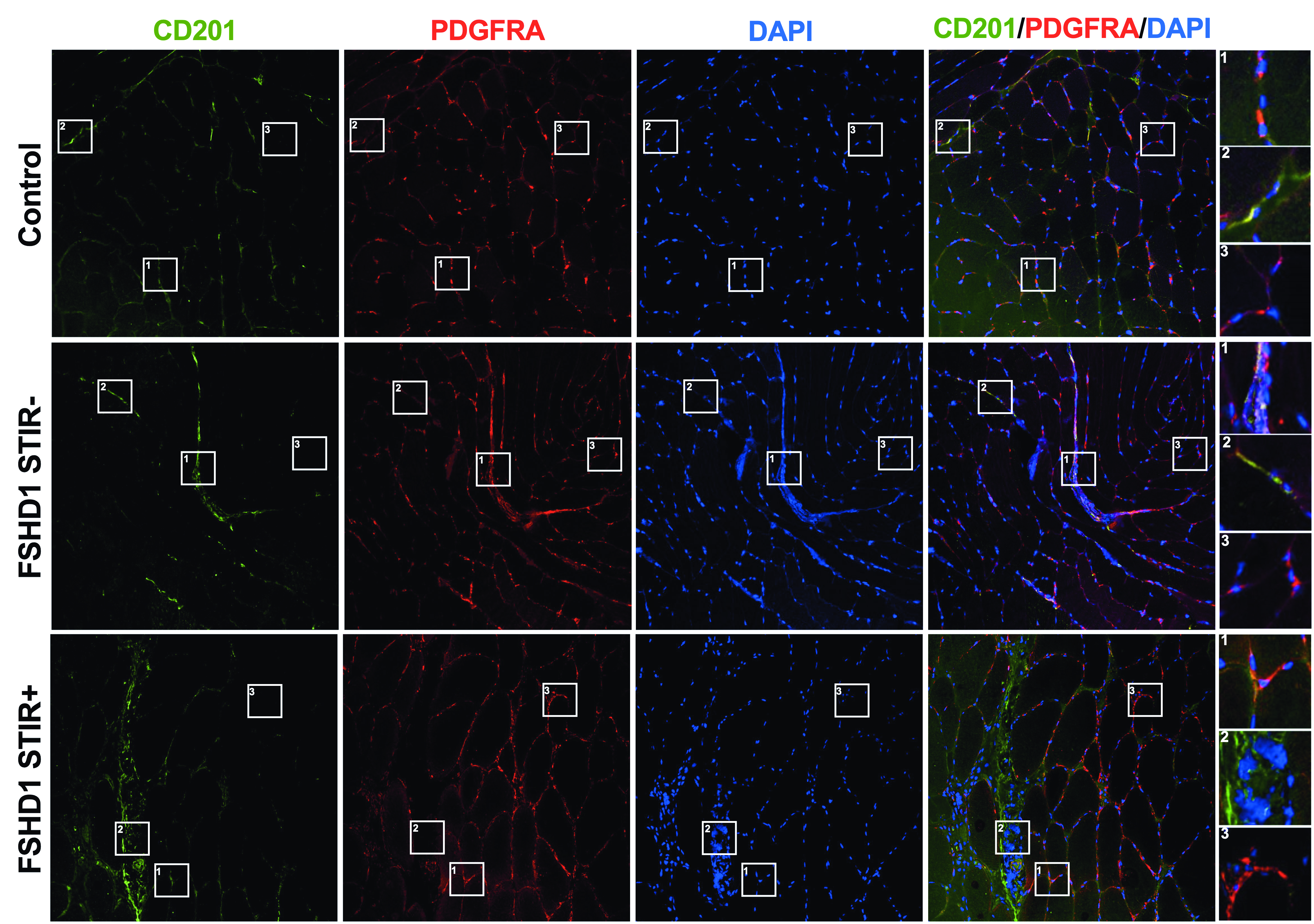

Supplement: Supplementary file 5 — Supplementary Figure S2 [file 41419_2022_5233_MOESM5_ESM.tif]

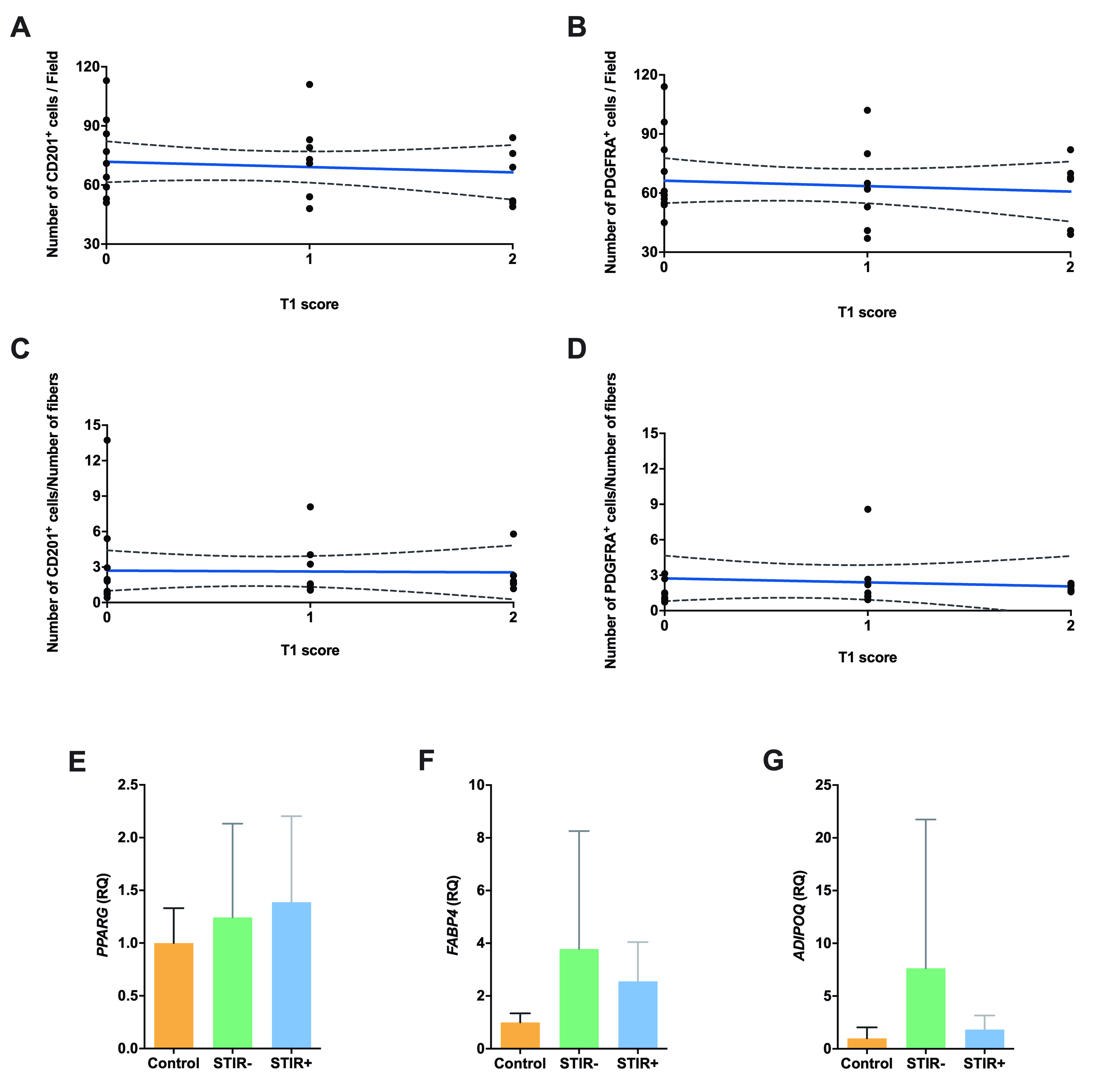

Supplement: Supplementary file 6 — Supplementary Figure S3 [file 41419_2022_5233_MOESM6_ESM.tif]
